# C-terminal domain phosphatase-like 1 (CPL1) is involved in floral transition in *Arabidopsis*

**DOI:** 10.1186/s12864-021-07966-8

**Published:** 2021-09-05

**Authors:** Chen Yuan, Jingya Xu, Qianqian Chen, Qinggang Liu, Yikai Hu, Yicheng Jin, Cheng Qin

**Affiliations:** 1 Research Centre for Plant RNA Signaling, College of Life and Environmental Sciences, Hangzhou Normal University, 311121 Hangzhou, China; 2 Division of Research and Development, Oriomics Inc, 310018 Hangzhou, China

**Keywords:** C-terminal domain phosphatase-like 1 (CPL1), Floral transition, *Arabidopsis*, Transcriptome, Vernalization pathway

## Abstract

**Background:**

RNA polymerase II plays critical roles in transcription in eukaryotic organisms. C-terminal Domain Phosphatase-like 1 (CPL1) regulates the phosphorylation state of the C-terminal domain of RNA polymerase II subunit B1, which is critical in determining RNA polymerase II activity. CPL1 plays an important role in miRNA biogenesis, plant growth and stress responses. Although *cpl1* mutant showes delayed-flowering phenotype, the molecular mechanism behind *CPL1*’s role in floral transition is still unknown.

**Results:**

To study the role of *CPL1* during the floral transition, we first tested phenotypes of *cpl1-3* mutant, which harbors a point-mutation. The *cpl1-3* mutant contains a G-to-A transition in the second exon, which results in an amino acid substitution from Glu to Lys (E116K). Further analyses found that the mutated amino acid (Glu) was conserved in these species. As a result, we found that the *cpl1-3* mutant experienced delayed flowering under both long- and short-day conditions, and CPL1 is involved in the vernalization pathway. Transcriptome analysis identified 109 genes differentially expressed in the *cpl1* mutant, with 2 being involved in floral transition. Differential expression of the two flowering-related DEGs was further validated by qRT-PCR.

**Conclusions:**

Flowering genetic pathways analysis coupled with transciptomic analysis provides potential genes related to floral transition in the *cpl1-3* mutant, and a framework for future studies of the molecular mechanisms behind *CPL1*’s role in floral transition.

**Supplementary Information:**

The online version contains supplementary material available at 10.1186/s12864-021-07966-8.

## Background

RNA polymerase II (Pol II) is a multiunit enzyme complex that plays critical roles in transcription in eukaryotic organisms. The C-terminal domain (CTD) of its largest subunit, RNA polymerase II subunit B1, recruits regulatory factors required to regulate transcription and RNA processing to RNA Pol II [1]. The Mediator complex integrates general transcription factors and gene-specific activators or repressors to this enzyme complex [2]. The CTD of RNA polymerase II subunit B1 consists of conserved heptad peptide repeats and their phosphorylation states are critical in determining RNA Pol II’s activity level [1, 3]. Many phosphatases play roles in regulating phosphorylation states of RNA polymerase II subunit B1 in yeast, plants and animals [1, 4–6]. In *Arabidopsis*, there are several CTD phosphatases [7–9]. Among them, CPL1 (C-terminal Domain Phosphatase-like 1) has been extensively studied in stress-response and gene-expression regulation [7, 9–13], and it specifically dephosphorylates the Ser5 residues of RNA Pol II CTD [14].

In *Arabidopsis*, the CTD phosphatase CPL1 plays an important role in modulating co-transcriptional pre-mRNA processing, thereby affecting growth and stress responses [15]. CPL1 is involved in responses to salt stress, iron deficiency, abscisic acid treatments and wounding [7, 9, 16, 17]. The mutants of *CPL1* have enhanced resistance to a leaf fungal pathogen (*Alternaria brassicicola*) and an aphid pest (*Myzus persicae*), which indicates that CPL1 also plays roles in pathogen and pest resistance [18].

CPL1 is essential for miRNA biogenesis [13, 19, 20]. The accuracy of processing primary miRNAs into mature miRNAs in plants is enhanced by SERRATE and HYPONASTIC LEAVES 1 (HYL1), and CPL1 interacts with both proteins [11]. Two serine residues of HYL1 are especially important for HYL1 functions, and hyperphosphorylated HYL1 is inactive [13]. The phosphorylation state of HYL1, and thus its activity level, is regulated by CPL1 [13]. CPL1-mediated HYL1 phosphorylation is regulated by Regulator of *CBF* Gene Expression 3 (RCF3) [19, 21, 22]. RCF3 interacts with CPL1 in the nucleus, and these interactions are essential to regulate the phosphorylation state of HYL1 [19]. The inactivation of RCF3 causes a phosphorylation shift of HYL1 towards the less active version [22].

Floral transition is one of the most important phase changes in flowering plants, which is regulated by both genetic and environmental factors. There are at least five flowering regulation pathways in *Arabidopsis*, including photoperiod pathway, vernalization pathway, autonomous pathway, gibberellin pathway and temperature pathway [23–27]. *MAF5* is a MADS-box transcription factor that represses floral transition [28], *MAF5* is the closest homolog of *FLOWERING LOCUS C* (*FLC*), an important repressor in the floral transition pathway [28–30]. There are five *FLC* homologs in *Arabidopsis*, *MAF1–5*, which, together with *FLC*, form a small family of closely related MADS-box transcription factors [31–33]. *MAF5* is normally repressed and its overexpression causes late-flowering [31]. *MAF5* is also involved in the prevention of precocious vernalization responses [34].

Many flowering-time regulators in the vernalization and autonomous pathways promote or inhibit flowering by directly regulating *FLC* and *MAF* expression levels [28, 35–37], including *FRIGIDA* (*FRI*). *FRI* is a major locus that determines the natural variation in *Arabidopsis* flowering time [38, 39], and it is responsible for the accelerated transition to flowering after vernalization in *Arabidopsis*. The plant-specific FRI possesses a coiled-coil domain and forms a large protein complex [38, 40]. FRI, FRIGIDA LIKE 1, FLC EXPRESSOR, FRIGIDA ESSENTIAL 1 and SUPPRESSOR OF FRIGIDA 4 form a complex known as FRIc that acts to promote *FLC* expression [40–43].The *cpl1* mutants undergo delayed flowering [18], but the molecular mechanism underlying *CPL1*’s involvement in floral transition is still largely unknown. Here, we found that a mutant harboring a point-mutation, *cpl1-3*, showed a delayed-flowering phenotype under both long-day and short-day conditions, and genetic pathway analyses revealed that CPL1 was involved in the vernalization pathway. To determine the molecular mechanism behind *CPL1’*s role in floral transition, a transcriptome analysis was performed. In total, 109 differentially expressed genes were found between wild-type and *cpl1-3* mutant seedlings at 9 days after germination. Among them, two DEGs were involved in floral transition. These results provide insights into genes potentially related to floral transition in the *cpl1-3* mutant and will aid in further studies of the molecular mechanisms underlying CPL1’s role in floral transition.

## Methods

### Plant materials and growth conditions

The mutants *co-9*, *ft-10*, Col:*FRI*^*SF2*^ (*FRI-Col*), *fld-3* and *fve-4* were all in the Col background [44, 45]. The *fpa-7* (SALK_138449), *fca-2* (SALK_057540) and *cpl1-3* (CS16351) seeds were bought from the Arabidopsis Biological Resource Center (http://www.arabidopsis.org/). All the plants were grown under long-day (16-h/8-h, light/dark) or short-day (8-h/16-h, light/dark) conditions, at 23 °C and a relative humidity of 75 %. The light intensity at the soil surface was 100 µmol m^− 2^ s^− 1^.

### Plasmid construction and transgenic plant generation

To construct *35 S:CPL1-3FLAG*, the *CPL1* coding sequence was amplified and then cloned into the binary vector *pCAMBIA1300-35 S:3FLAG*. The primers used for plasmid construction are listed in Additional file 1.

Transgenic plants were generated through *Agrobacterium tumefaciens*-mediated transformation using the floral-dipping method. The *cpl1-3* mutants used for transformation were approximately 4 weeks old with plenty of inflorescences. Developing floral tissues were dipped into an *Agrobacterium* solution containing 5 % sucrose and Silwet-77 (500 µL L^− 1^). Transformants containing *35 S:CPL1-3FLAG* were selected on MS medium supplemented with hygromycin (30 mg L^− 1^). Two independent homozygous T3 lines with hygromycin resistance were chosen for further studies.

### Cleaved Amplified Polymorphic Sequences (CAPS) Analysis

A 610-bp DNA fragment of WT or the *cpl1-3* mutant was amplified using the following primers: Forward, 5′-TCTGGCGAGAGGTGTCC-3′/Reverse, 5′-GCTGAAACCCGTCAATCTTAT-3′. PCR was carried out as follows: 40 cycles of 95 °C for 30 s, 58 °C for 30 s and 72 °C for 1 min. Then, the PCR products were digested by Sac I and separated on 1 % agarose-TAE gels.

### Flowering-time measurement

The flowering times of the plants were measured by assessing the numbers of rosette leaves and the number of days when the first flowers appeared.

### Transcriptomic analyses

Total RNA was isolated using an RNAprep Pure Plant Kit (TIANGEN, Beijing, China), and then, DNase I was added to eliminate genomic and plastid DNA. The isolated total RNA was analyzed using a NanoDrop and Agilent 2100 Bioanalyzer (Thermo Fisher Scientific, MA, USA). mRNA was purified using Oligo (dT) magnetic beads and then sheared into small fragments. The first-strand cDNA was reverse transcribed using random hexamer primers, followed by second-strand cDNA synthesis. Then, an A-Tailing Mix and RNA Index Adapters were added. The resultant cDNA fragments were amplified by PCR, and then dissolved in EB solution. The double-stranded PCR products were heat denatured to produce the final library. The sequencing was performed on a BGIseq500 platform (BGI-Shenzhen, China). The transcriptome data were filtered and analyzed in accordance with a previous paper [46]. Differential expression analyses were performed using the following criteria: |log2(-fold change) | > 1 and Q value < 0.05.

### qRT-PCR

In total, 1 µg RNA was reverse transcribed using a FastKing gDNA Dispelling RT SuperMix kit (TIANGEN) in accordance with the manufacturer’s instructions. The qRT-PCR was performed using an UltraSYBR Mixture (with ROX; CWBio, Beijing, China) and the CFX96 real-time PCR detection system (Bio-Rad, CA, USA). The expression of *TUBULIN 2* (*TUB*2) was used as an internal control. Error bars denote SD of three biological replicates. All the primers used for qRT-PCR are listed in Additional file 1.

### Statistical analysis

The experimental data were analyzed using two-tailed paired Student’s *t* tests with SPSS 12.0 software.

## Results

### Loss of CPL1 function delays flowering in *Arabidopsis*

**Fig. 1:**
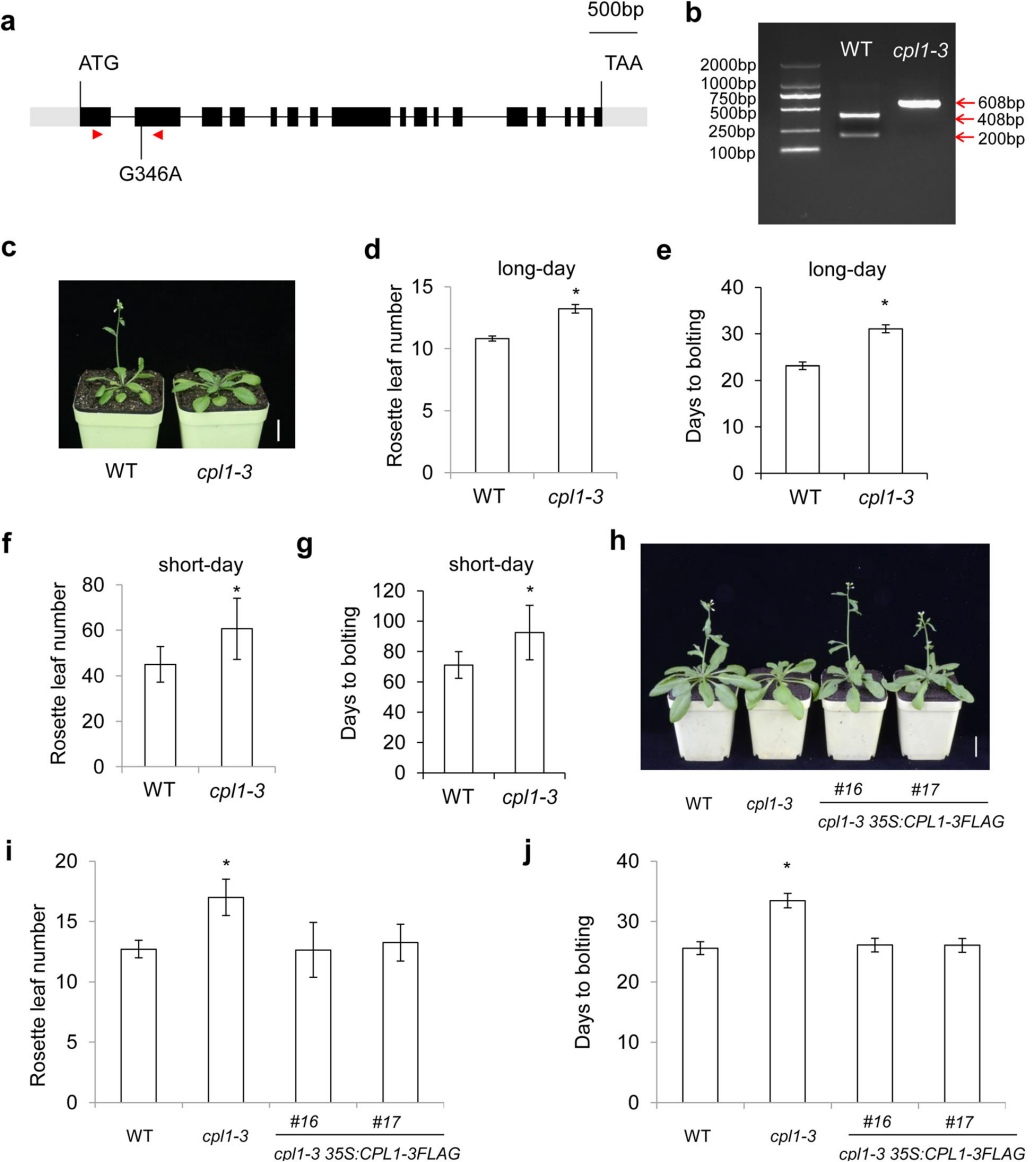
*CPL1* regulates flowering time in *Arabidopsis*. **a** The structure of the CPL1 coding region. Black boxes, gray boxes and black lines represent exons, untranslated regions and introns, respectively. The point mutation is shown below. Red arrowheads indicate the positions of the primers used in Fig. 1b. **b** A cropped gel of the CAPS analysis of wild-type and the *cpl1-3* mutant. Genomic DNA of wild-type (WT) and the *cpl1-3* mutant were amplified using CAPS markers listed in Additional file 1, and then, the PCR products were digested with Sac I. **c ***cpl1-3* mutant shows delay-flowering phenotype under long-day conditions. Scale bar: 2 cm. **d and e** Rosette leaf numbers (**d**) and days to bolting (**e**) of the *cpl1-3* mutant grown under long-day conditions. **f and g** Rosette leaf numbers (**f**) and days to bolting (**g**) of the *cpl1-3* mutant grown under short-day conditions. Values are representative of at least 15 plants showing specific genotypes. Asterisks indicate significant differences between WT and the *cpl1-3* mutant in flowering time (Student’s *t * test, *P* < 0.05). **h** *cpl1-1-3 35 S:CPL1-3FLAG* exhibited a flowering time comparable to that of WT plants under long-day conditions. Scale bar: 2 cm. **i and j** Rosette leaf numbers (**i**) and days to bolting (**j**) of *cpl1-1-3 35 S:CPL1-3FLAG* grown under long-day conditions. Values are representative of at least 15 plants showing specific genotypes. Asterisks indicate significant differences between WT and the *cpl1-3* mutant in flowering time (Student’s *t* test, *P* < 0.05)

CPL1 regulates flowering time in *Arabidopsis* [18], but the molecular mechanism underlying *CPL1*’s role in floral transition is still unknown. To elucidate this, we used the *cpl1-3* mutant harboring a point-mutation to examine the flowering phenotype [17]. The *cpl1-3* mutant contains a G-to-A transition in the second exon (Fig. 1a), which results in an amino acid substitution from Glu to Lys (E116K) (Additional file 2) and leads to the loss of a Sac I site in the *CPL1* gene (Fig. 1b). We then analyzed CPL protein sequence of Arabidopsis and other different species, and found that the mutated amino acid (Glu) was conserved in these species (Additional file 3). The *cpl1-3* mutant displayed a delayed-flowering phenotype under both long- and short-day conditions (Fig. 1d–g), suggesting that *CPL1* acts as an activator in floral transition.

To confirm the loss of CPL1 function was responsible for the delayed-flowering phenotype of the *cpl1-3* mutant, we transformed the *cpl1-3* mutant with a construct containing the coding sequence of *CPL1* driven by the constitutive cauliflower mosaic virus 35 S promoter. Two independent *cpl1-1-3 35 S:CPL1-3FLAG* transgenic lines exhibited comparable flowering times to WT plants (Fig. 1 h-j; Additional file 4), indicating that CPL1 was responsible for the flowering phenotype of the *cpl1-3* mutant and that excess amounts of *CPL1* do not further accelerate flowering.

We further analyzed the *CPL1* expression in different tissues of WT plants. The qRT–PCR results showed that *CPL1* was mainly expressed in leaves (Additional file 5).

### *CPL1* is involved in the vernalization pathway

**Fig. 2:**
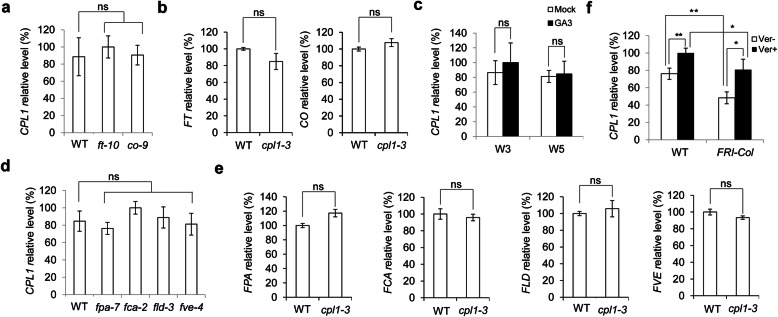
*CPL1* expression is regulated by the vernalization pathway. **a ***CPL1* expression in photoperiod-pathway mutants at 9 DAG. **b ***FT* and *CO* expression in WT and *cpl1-3* mutant seedlings. **c ***CPL1* expression after a gibberellin treatment. The WT seedlings were grown under short-day conditions for 2 weeks and then treated with 100 µM gibberellic acid or 0.1 % ethanol weekly. After 3 weeks (W3) and 5 weeks (W5), samples were collected for further analyses. **d ***CPL1* expression in autonomous-pathway mutants at 9 DAG. **e ***FPA*, *FCA*, *FLD* and *FVE* expression in WT and *cpl1-3* mutant seedlings. **f ***CPL1* expression after the vernalization treatment. The seeds were vernalized at 4 °C for 8 weeks, and 9-day-old seedlings were collected for further analyses. Asterisks indicate significant differences (Student’s *t* test, *P < 0.05, **P < 0.01, “ns” indicates statistically not siginificant)

Because CPL1 regulates flowering time in *Arabidopsis*, we examined whether CPL1 had roles in the flowering-related genetic pathways. The *CPL1* expression level did not significantly change in photoperiod pathway mutants (Fig. 2a), *FT* and *CO* expression were also not changed in WT and *cpl1-3* mutant seedlings (Fig. 2b), and the *cpl1-3* mutant flowered late, compared with WT, under both long- and short-day conditions (Fig. 1c–e), indicating that *CPL1* may not be involved in the photoperiod pathway. A gibberellin treatment did not alter the *CPL1* expression level (Fig. 2c), indicating that *CPL1* may not be involved in the gibberellin pathway. The generation of autonomous pathway mutants did not significantly disrupt the *CPL1* expression level (Fig. 2d), while *FPA*, *FCA*, *FLD* and *FVE* expression were all consistent in WT and *cpl1-3* mutant seedlings (Fig. 2e), indicating that *CPL1* may not be involved in the autonomous pathway. We then used *FRI-Col* seedlings, which contained *FRIGIDA* (*FRI*) in the Col background and elevated *FLC* transcript level, for further analyses [42]. However, after a vernalization treatment, the *CPL1* expression level was elevated in both WT and *FRI-Col* seedlings (Fig. 2f), suggested that CPL1 may play roles in the vernalization pathway. Furthermore, the *CPL1* expression level declined in *FRI-Col* seedlings compared with in WT seedlings independent of the vernalization treatment (Fig. 2f). This indicated that FRI repressed *CPL1* expression. The results suggest that CPL1 may be involved in the vernalization pathway.

### Transcriptome analysis and DEG Identification between WT and the *cpl1-3* Mutant

To identify the downstream flowering-time regulators that are responsible for the function of *CPL1* in floral transition, RNA-seq analyses were performed. Total RNA isolated from WT and *cpl1-3* mutant seedling at 9 DAG were used to construct libraries. Three biological replicates were used, and six libraries were constructed for transcriptome sequencing. The detailed information on the RNA-seq reads used for constructing the six libraries are shown in Additional file 6. Briefly, 271.97 million raw reads were generated. After qualifying and filtering, approximately 267.24 million clean reads (approximately 98.3 %), comprising 40.08 Gb of sequence data, were used for further studies. Over 95.93 % of the clean reads had quality scores at the Q20 level, and over 86.69 % of the clean reads have quality scores at the Q30 level.

**Fig. 3:**
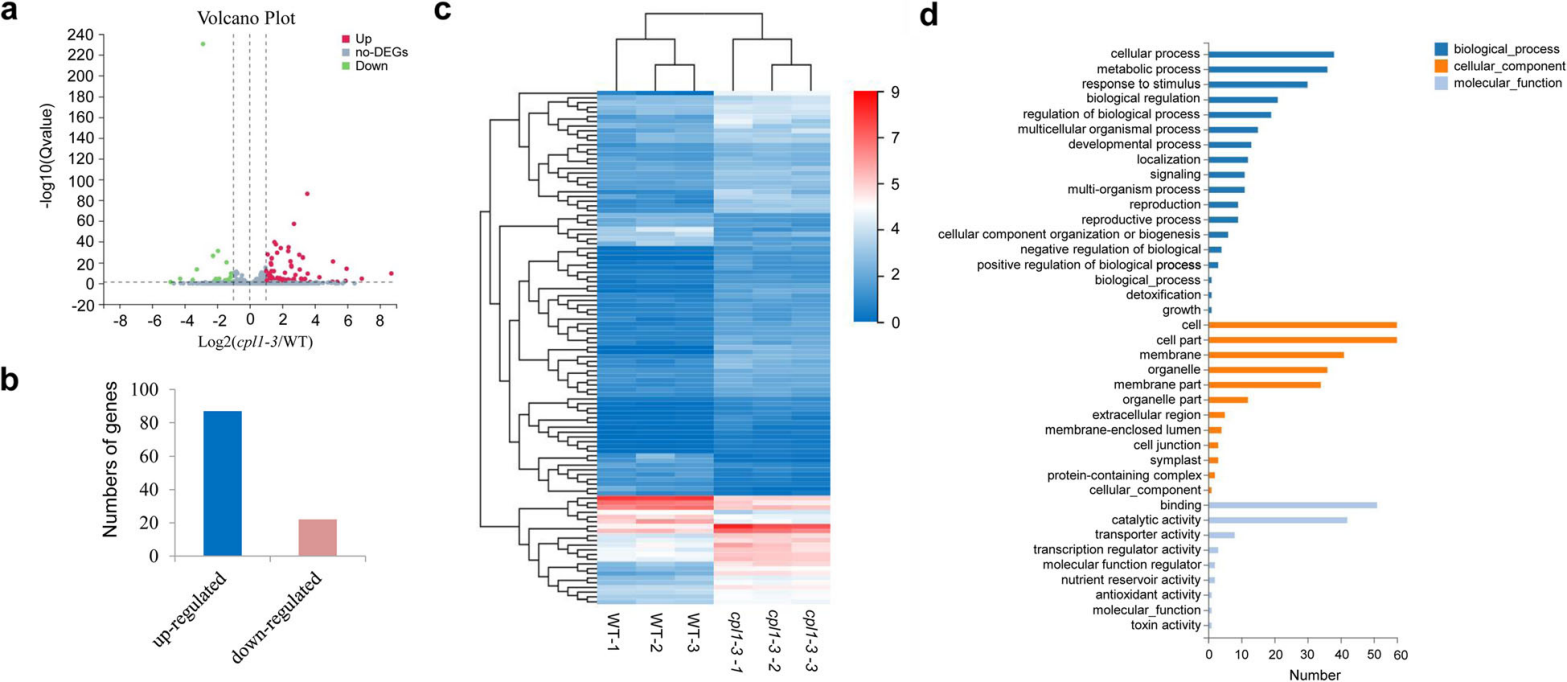
Transcriptional profiles in WT and *cpl1-3* mutant seedlings. **a** Significance analysis of all the DEGs between WT and *cpl1-3* mutant seedlings displayed using a volcano plot. **b** The numbers of genes that were up- and down-regulated between WT and *cpl1-3* mutant seedlings. **c** Expression profiles of the differentially expressed genes between WT and *cpl1-3* mutant seedlings shown using a heatmap. **d** GO enrichment analysis of DEGs between WT and *cpl1-3* mutant seedlings

As a result, 109 DEGs between WT and the *cpl1-3* mutant meeting the criteria |log2(-fold change)| > 1 and Q value < 0.05 (Fig. 3a) were identified and analyzed. Among them, 87 DEGs were up-regulated and 22 DEGs were down-regulated (Fig. 3b). A heatmap of the DEG expression profiles clearly revealed that the samples were separated into two clusters, indicating that the three biological replicates of WT and *cpl1-3* mutant were highly repeatable (Fig. 3c). An analysis of the biological functions of these DEGs was performed. For the GO classification, the top five largest GO terms in biological process were “cellular process”, “metabolic process”, “response to stimulus”, “biological regulation”, and “regulation of biological process”; in cellular component, the top five largest GO terms were “cell”, “cell part”, “membrane”, “organelle” and “membrane part”; and in molecular function, “binding”, “catalytic activity” were the two largest GO terms (Fig. 3d).

### Identification of Flowering-time-related DEGs and Validation of RNA-seq Data

To investigate the molecular mechanisms underlying *CPL1’*s role in floral transition, we analyzed the DEGs to determine which were involved in floral transition and responsible for the flowering phenotype of the *cpl1-3* mutant. Among the 109 DEGs between WT and the *cpl1-3* mutant, 2 DEGs were involved in flowering-related genetic pathways. The expression level of *MADS AFFECTING FLOWERING 5* (*MAF5*) was up-regulated, whereas that of *TWIN SISTER OF FT* (*TSF*) was down-regulated in *cpl1-3* mutant seedlings (Additional file 7).

**Fig. 4:**
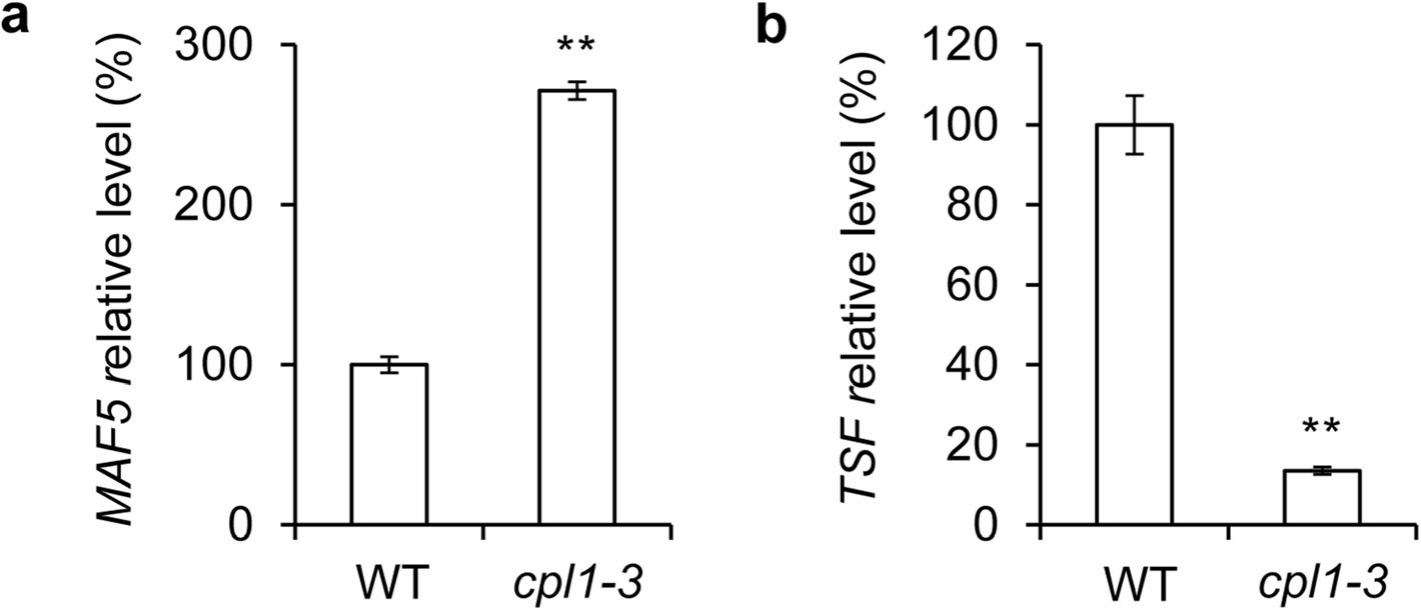
Validation of flowering-time-related DEGs by qRT-PCR. Expression levels of MAF5 (**a**) and TSF (**b**) were determined by qRT-PCR. Error bars indicate SDs of three biological replicates. Asterisks indicate significant differences (Student’s *t* test, **P < 0.01)

To validate the DEG expression levels identified by RNA-seq, qRT-PCR was performed. As shown in Fig. 4a, b, the qRT-PCR results were consistent with RNA abundance levels inferred from the RNA-seq experiments. This result suggests that the RNA-seq data are reliable.

## Discussion

CPL1 plays critical roles in transcriptional regulation, and is thus involved in miRNA biogenesis, plant growth and stress responses. In this study, we found that the loss of *CPL1* function resulted in a delayed-flowering phenotype in *Arabidopsis* (Fig. 1). To investigate the molecular mechanisms underlying *CPL1’*s role in floral transition, transcriptomic analyses between WT and *cpl1-3* mutant seedlings were performed. As a result, 109 DEGs were revealed. Among them, two DEGs, *MAF5* and *TSF*, functioned in floral transition (Fig. 4; Additional file 7).

In this study, we found that FRI repressed the expression of *CPL1*, and a vernalization treatment induced the expression of *CPL1* in both WT and *FRI-Col* seedlings (Fig. 2f). These results indicated that CPL1 plays roles in the vernalization pathway and acts downstream of FRI. This, together with the up-regulated expression of *MAF5* in *cpl1-3* mutant seedlings (Fig. 4; Additional file 7), led us to speculate that *FRI* may upregulate *MAF5* expression and that responses to vernalization may require functional CPL1.

CPL1 is a phosphatase that can regulate the phosphorylation state of many proteins, including RNA polymerase II subunit B1 and HYL1 [13, 14]. In this study, as the *MAF5* expression was up-regulated in *cpl1-3* mutant seedlings, indicated that CPL1 may interact with other proteins which regulate the expression of *MAF5*, and then regulated the phosphorylation state of these proteins and thus their activity levels in regulating *MAF5* expression. Further studies should focus on screening CPL1-interacting proteins which may be response for the regulation of *MAF5* expression.

In *Arabidopsis*, *TSF* is the closest homolog of *FLOWERING LOCUS T* (*FT*), sharing an approximately 82 % amino acid sequence identity [47, 48]. *TSF* and *FT* expressed in rootstock plants accelerate the flowering of grafted *tsf* or *ft* mutant scions, indicating that TSF and FT act through a similar mechanism of protein movement towards the shoot apex, which triggers flowering. The effect of *TSF* on triggering flowering in mutant scions was weaker than that of *FT* [49], perhaps because TSF is less mobile than FT. In *Arabidopsis*, FLC directly suppresses the expression of floral pathway integrators, such as *FT* [50, 51]. In this study, the *MAF5* expression was up-regulated, while *TSF* expression was down-regulated, in *cpl1-3* mutant seedlings, we supposed that MAF5 may also suppress the expression of *TSF*, but we also cannot exclude the possibility that CPL1 directly regulates the expression of *TSF* by interacting with other proteins, further studies should focus on this.

## Conclusions

In summary, a transcriptome analysis was performed between wild-type and *cpl1-3* mutant seedlings at 9 DAG. Through bioimformatics mining, 109 differentially expressed genes were identified, with two genes, *MAF5* and *TSF*, were involved in floral transition. Differential expression of the two flowering-related DEGs was further validated by qRT-PCR. Furthermore, *CPL1* expression decreased in *FRI* seedlings, whereas a vernalization treatment induced *CPL1* expression (Fig. 2f). Considering that the expression level of *MAF5*, the closest homolog of *FLC*, increased in *cpl1-3* seedlings (Fig. 4; Additional file 7), and that the *cpl1-3* mutant displayed a delayed-flowering phenotype under both long- and short-day conditions (Fig. 1c–g), we propose that *FRI* may regulate *MAF5* expression through *CPL1* and may subsequently suppress downstream *TSF* to delay flowering. We envisage that further studies on how *CPL1* impacts on *FRI* to regulate *MAF5* expression will deepen our knowledge into the vernalization pathway in control of flowering.

## Supplementary Information

**Additional file 1.** The primers used in this study.

**Additional file 2. **Alignment of WT and mutant CPL1 protein sequences.

**Additional file 3. **Alignment of CPL protein sequence of Arabidopsis and other different species. Red rectangle indicates the position of mutated amino acid (Glu).

**Additional file 4. **The*CPL1*expression level in independent *CPL1-*overexpression lines. Seedlings were collected at 9 DAG. The levels of gene expression normalized to *TUB2* expression are shown as relative values to that of WT set at 1. Error bars indicate SD of three biological replicates.

**Additional file 5. **The*CPL1* expression level in various tissues of WT plants as assessed by qRT-PCR. JRL, juvenile rosette leaves; Rt, roots; ARL, adult rosette leaves; CL, cauline leaves; FL, flowers; St, inflorescence stems; Sil, siliques. Expression levels are shown as relative values to the maximal level set at 100%. Error bars indicate SD of three biological replicates.

**Additional file 6. **The detailed information on raw reads from different samples.

**Additional file 7. **The list of 109 DEGs between WT and the *cpl1-3* mutant.

**Additional file 8. **The list of vernalization related genes between WT and the *cpl1-3* mutant.

**Additional file 9. **The original, full-length gel displayed in Fig. 1b.

## Data Availability

The datasets supporting the conclusions of this article are available in the NCBI Short Read Archive with accession number PRJNA699075 (https://www.ncbi.nlm.nih.gov/bioproject/?term=prjna699075).

## Abbreviations

CAPS: Cleaved amplified polymorphic sequences
CPL1: C-terminal Domain Phosphatase-like 1
CTD: C-terminal domain
DAG: Days after germination
DEGs: Differentially expressed genes
FLC: FLOWERING LOCUS C
FRI: FRIGIDA
FT: FLOWERING LOCUS T
GA: Gibberellin
HYL1: HYPONASTIC LEAVES 1
LD: Long-day
MAF5: MADS AFFECTING FLOWERING 5
qRT-PCR: quantitative real-time PCR
Pol II: RNA polymerase II
RCF3: Regulator of *CBF* Gene Expression 3
RNA-seq: RNA sequencing
SD: Short-day
TSF: TWIN SISTER OF FT
TUB2: TUBULIN 2
WT: wild-type
