# Supplementary material for: C-terminal domain phosphatase-like 1 (CPL1) is involved in floral transition in Arabidopsis
Source: BMC Genomics. 2021 Sep 5;22:642. doi: 10.1186/s12864-021-07966-8 (PMC8418720; doi:10.1186/s12864-021-07966-8)
Supplement: Supplementary file 1 — Additional file 1. The primers used in this study. [file 12864_2021_7966_MOESM1_ESM.docx]

**Table S3. List of primers used in this study**

**Primers for plasmid construction**

| **Construct name** | **Sequence (5’-3’)** |
| --- | --- |
| *35S: CPL1-3FLAG* | tggagagaacacgggggacgATGTATAGTAATAATAGAGT |
|  | gtcgactctagaggatccccgAGAGTATCTTCCCGAAGATG |

**Primers for quantitative real-time PCR**

| **Gene name** | **Sequence (5’-3’)** |
| --- | --- |
| *CPL1* | CGTCCTTGTTTCTGGGCATTT |
|  | CGTTGTTTATTCTCCGCTGAA |
| *MAF5* | GATGGAGCTTGTGAAGAACCTTCAGG |
|  | CAGCCGTTGATGATTGGTGGTTACTTG |
| *TSF* | CTTGATCCTTTCACGAGGTTGG |
|  | GGCAGTTGAAGTAAGAGGCA |
| *Tub2* | ATCCGTGAAGAGTACCCAGAT |
|  | AAGAACCATGCACTCATCAGC |

**Primers for genotyping assays**

| **Gene name** | **Sequence (5’-3’)** |
| --- | --- |
| *cpl1-3* | TCTGGCGAGAGGTGTCC |
|  | GCTGAAACCCGTCAATCTTAT |
