# Supplementary figures and images for: C-terminal domain phosphatase-like 1 (CPL1) is involved in floral transition in Arabidopsis
Source: BMC Genomics. 2021 Sep 5;22:642. doi: 10.1186/s12864-021-07966-8 (PMC8418720; doi:10.1186/s12864-021-07966-8)

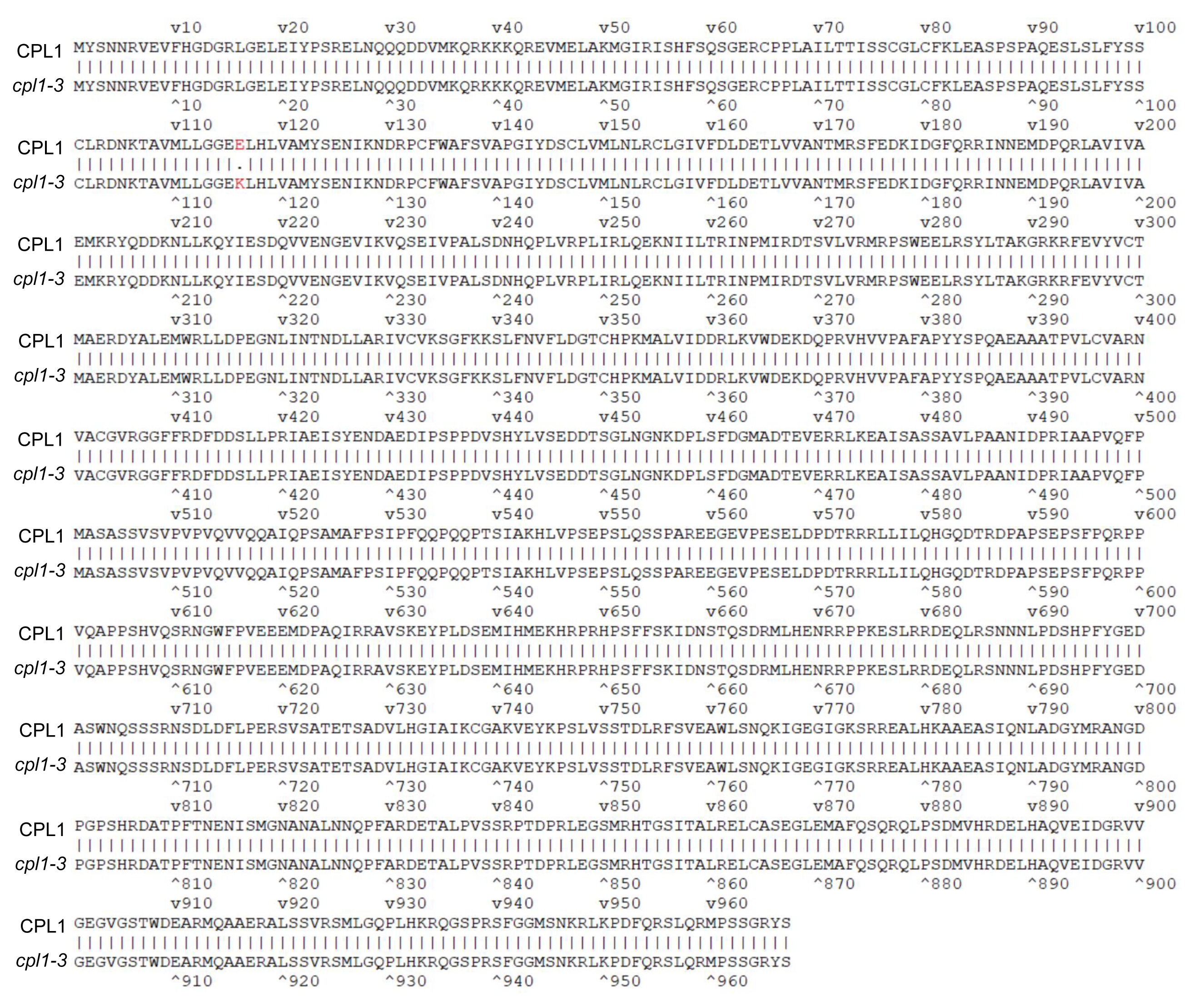

Supplement: Supplementary file 2 — Additional file 2. Alignment of WT and mutant CPL1 protein sequences. [file 12864_2021_7966_MOESM2_ESM.tif]

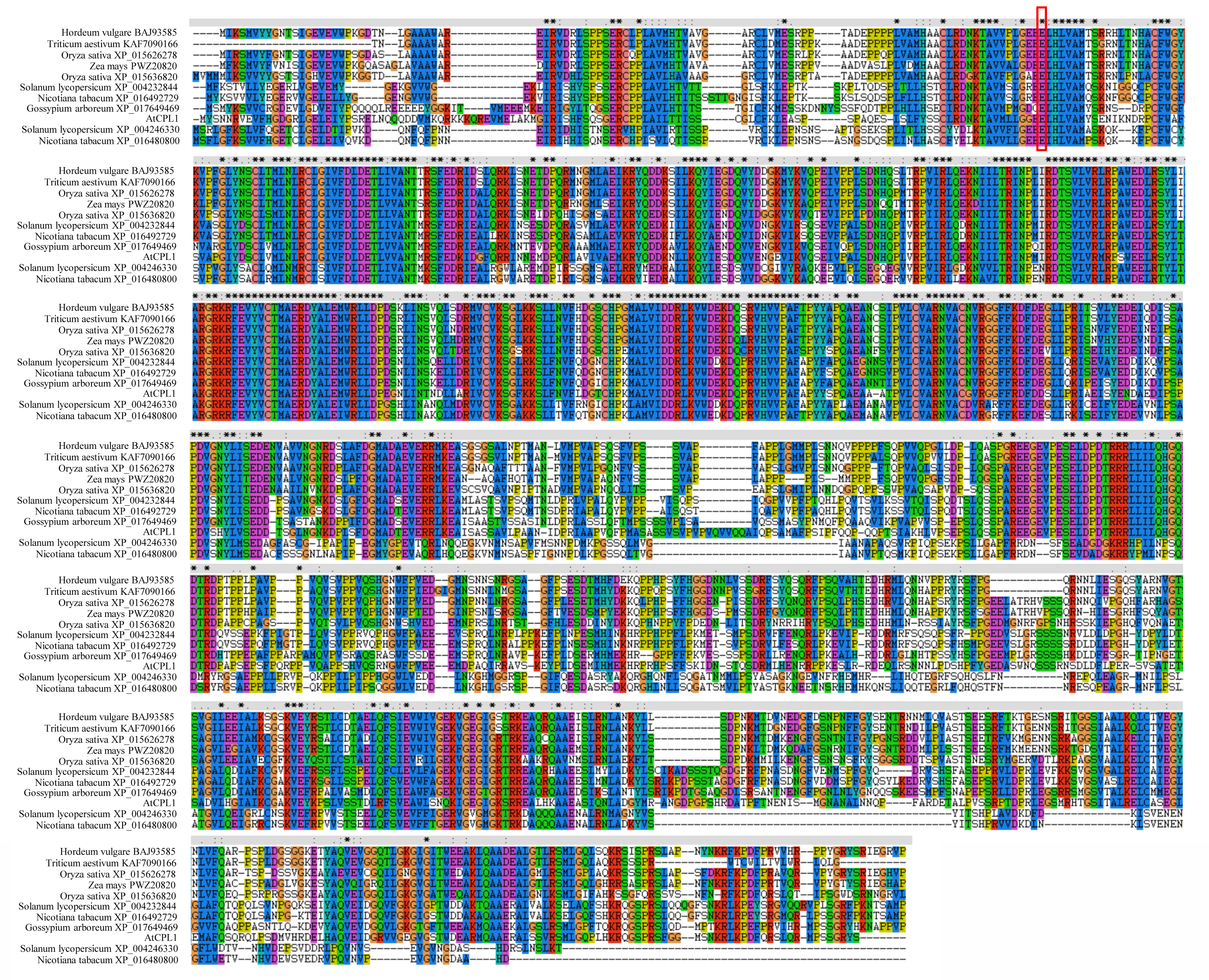

Supplement: Supplementary file 3 — Additional file 3. Alignment of CPL protein sequence of Arabidopsis and other different species. Red rectangle indicates the position of mutated amino acid (Glu). [file 12864_2021_7966_MOESM3_ESM.jpg]

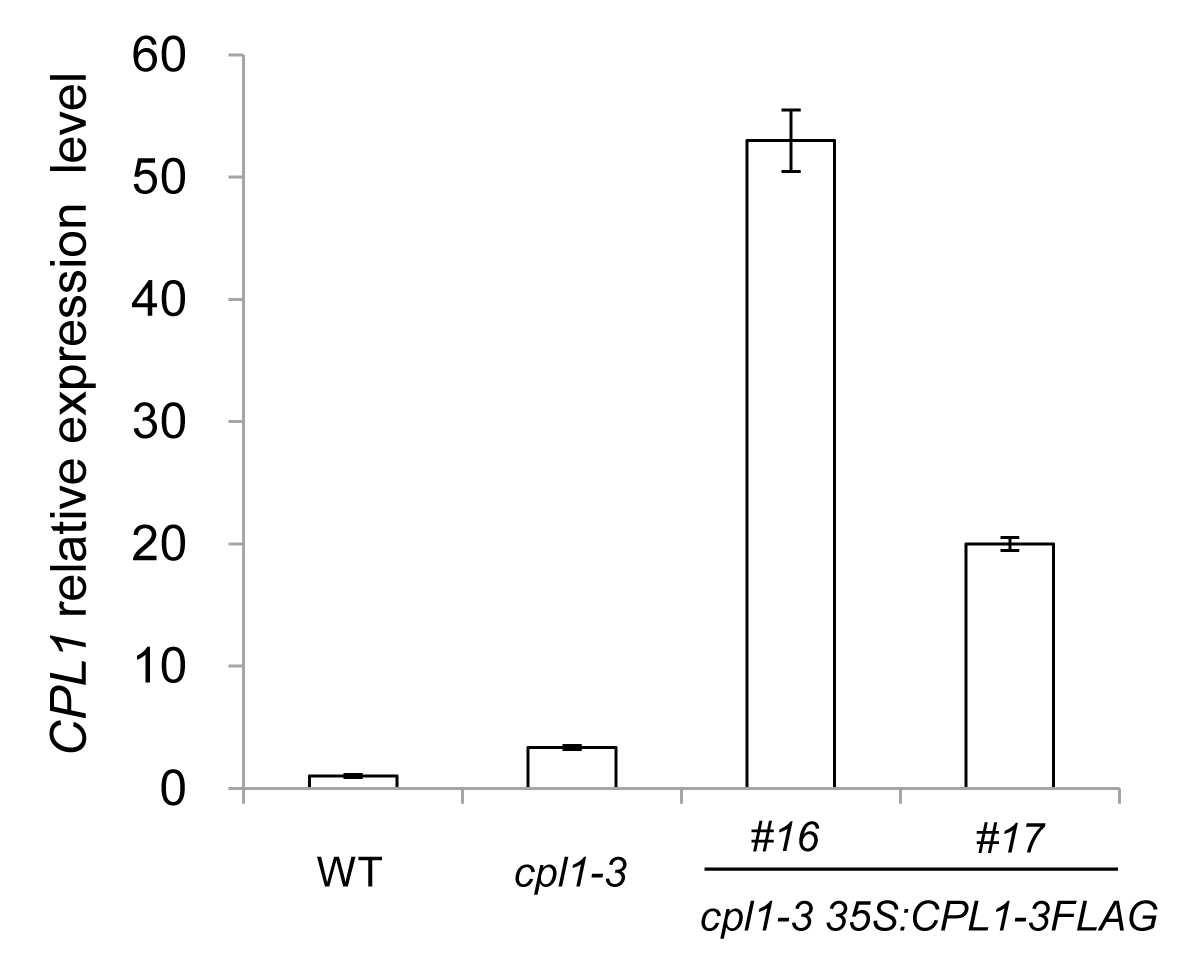

Supplement: Supplementary file 4 — Additional file 4. TheCPL1expression level in independent CPL1-overexpression lines. Seedlings were collected at 9 DAG. The levels of gene expression normalized to TUB2 expression are shown as relative values to that of WT set at 1. Error bars indicate SD of three biological replicates. [file 12864_2021_7966_MOESM4_ESM.tif]

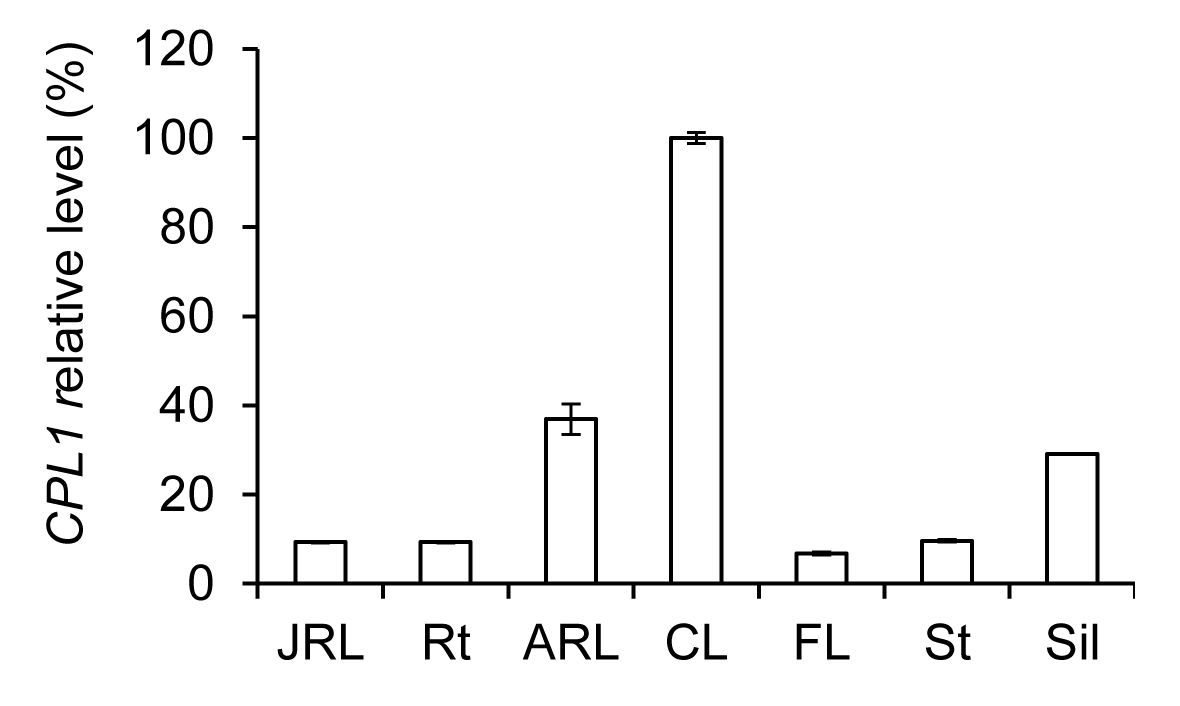

Supplement: Supplementary file 5 — Additional file 5. TheCPL1 expression level in various tissues of WT plants as assessed by qRT-PCR. JRL, juvenile rosette leaves; Rt, roots; ARL, adult rosette leaves; CL, cauline leaves; FL, flowers; St, inflorescence stems; Sil, siliques. Expression levels are shown as relative values to the maximal level set at 100%. Error bars indicate SD of three biological replicates. [file 12864_2021_7966_MOESM5_ESM.tif]

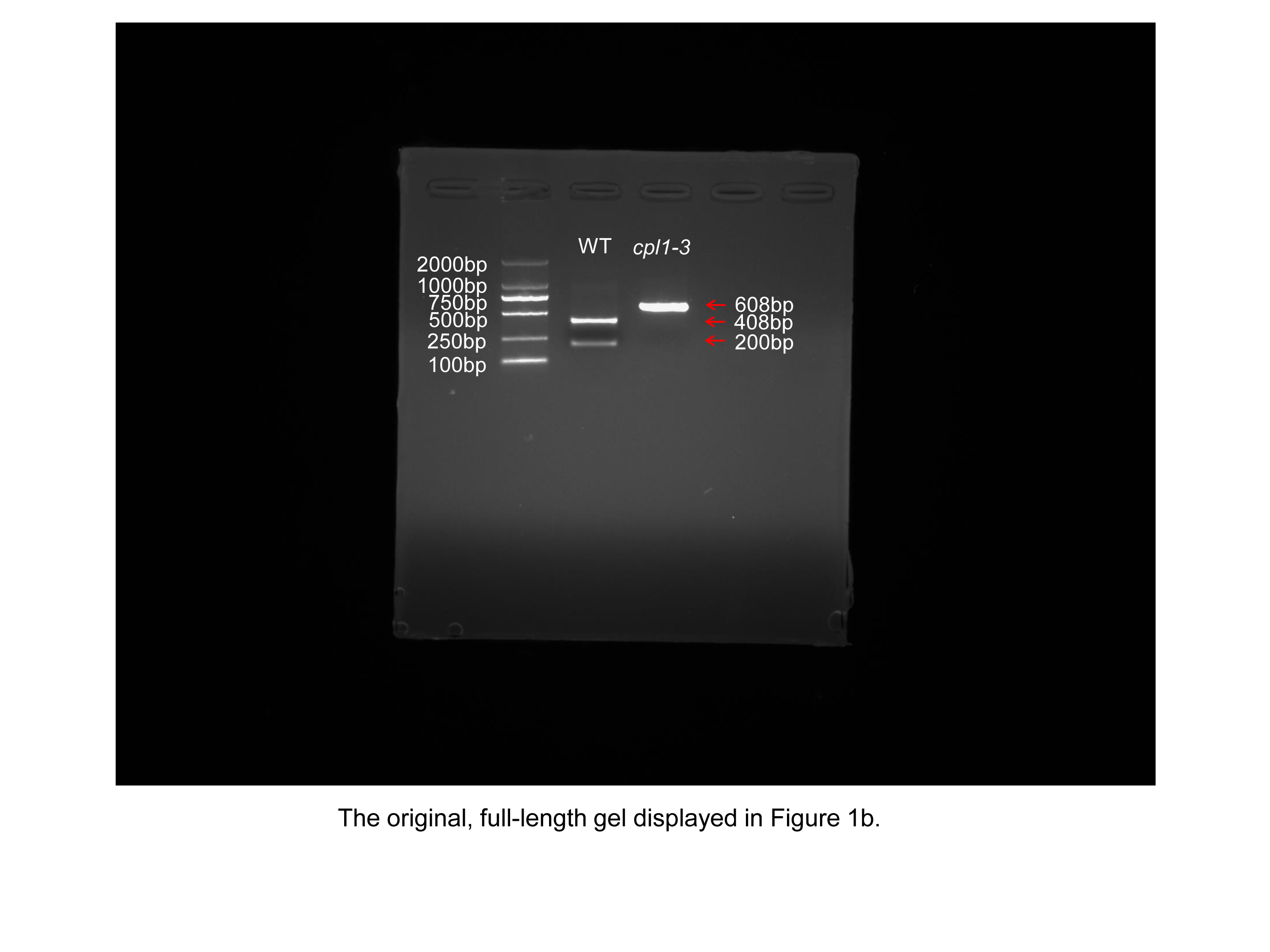

Supplement: Supplementary file 9 — Additional file 9. The original, full-length gel displayed in Fig. 1b. [file 12864_2021_7966_MOESM9_ESM.tif]
